# Progress in Research on the Role of Flavonoids in Lung Cancer

**DOI:** 10.3390/ijms20174291

**Published:** 2019-09-02

**Authors:** Oana Zanoaga, Cornelia Braicu, Ancuta Jurj, Alexandru Rusu, Rares Buiga, Ioana Berindan-Neagoe

**Affiliations:** 1 Research Center for Functional Genomics and Translational Medicine, Iuliu Hatieganu University of Medicine and Pharmacy, 23 Marinescu Street, 40015 Cluj-Napoca, Romania; 2 Biozoon GmbH, D-27572 Bremerhaven, Germany; 3 Department of Pathology, “Prof. Dr. Ion Chiricuta” Oncology Institute, 400015 Cluj-Napoca, Romania; 4 Department of Pathology Iuliu Hatieganu, University of Medicine and Pharmacy, 400015 Cluj-Napoca, Romania; 5 MEDFUTURE-Research Center for Advanced Medicine, Iuliu Hatieganu University of Medicine and Pharmacy, Cluj-Napoca, Romania, 23 Marinescu Street, 40015 Cluj-Napoca, Romania; 6 Department of Functional Genomics and Experimental Pathology, The Oncology Institute “Prof. Dr. Ion Chiricuta”, Republicii 34th street, 400015 Cluj-Napoca, Romania

**Keywords:** flavonoids, lung cancer, cellular signaling pathways

## Abstract

Lung cancer is the leading cause of cancer deaths worldwide. Therefore, for the prevention, diagnosis, prognosis and treatment of lung cancer, efficient preventive strategies and new therapeutic strategies are needed to face these challenges. Natural bioactive compounds and particular flavonoids compounds have been proven to have an important role in lung cancer prevention and of particular interest is the dose used for these studies, to underline the molecular effects and mechanisms at a physiological concentration. The purpose of this review was to summarize the current state of knowledge regarding relevant molecular mechanisms involved in the pharmacological effects, with a special focus on the anti-cancer role, by regulating the coding and non-coding genes. Furthermore, this review focused on the most commonly altered and most clinically relevant oncogenes and tumor suppressor genes and microRNAs in lung cancer. Particular attention was given to the biological effect in tandem with conventional therapy, emphasizing the role in the regulation of drug resistance related mechanisms.

## 1. Introduction

Lung cancer is one of the most dangerous types of cancer for both men and women with an increasing number of deaths each year and a survival rate lower than other types of malignancies, that is, with overall 5-year survival rates of 10–20% in most countries [1,2]. According to GLOBOCAN 2018, it was estimated that lung cancer is the most commonly diagnosed cancer (11.6% of the total cases) and the leading cause of cancer death (18.4% of the total cancer deaths) accounting for 2.1 million new cases and 1.8 million deaths in 2018 [3].

In spite of the progress in treatment options involving surgery, radiation, chemotherapy and specific targeted therapies, the prognosis remains unsatisfactory due to the late diagnosis, being related to the appearance of metastatic disease [4,5]. A considerable number of genomic studies associate lung cancer with *TP53* (tumor protein 53), *PTEN* (phosphatase and tensin homolog deleted on chromosome 10) and *PI3K/Akt* (phosphatidylinositol-3-kinase) mutations [6], RTK alteration (receptor of tyrosine kinase like *EGFR* (epidermal growth factor receptor), *MET*, *ROS1* (reactive oxygen species), *ALK* (anaplastic lymphoma kinase) and *RET* (proto-oncogene tyrosine-protein kinase receptor) [7]. These genomic alterations affect multiple cellular functions, including the cell growth, differentiation, proliferation, survival, motility, invasion and intracellular trafficking. The association of activating *RTK* mutations, giving enlarged sensitivity and disease response to RTK inhibitors, permitted the development of new approaches for the personalized treatment of lung cancer. The comprehensive epigenetic, genomic and molecular characterization of lung cancer has guided the identification of novel targeted therapies based on natural or synthetic small molecules [5,8]. The major risk factors for lung cancer, like smoking tobacco and exposure to chemical carcinogens, are sustained by an unhealthy inflammatory diet (low consumption of fruits and vegetables and high salt consumption [9]). It is known that the Mediterranean diet rich in natural phytochemicals are related to a reduced risk of lung cancer [10].

**Figure 1:**
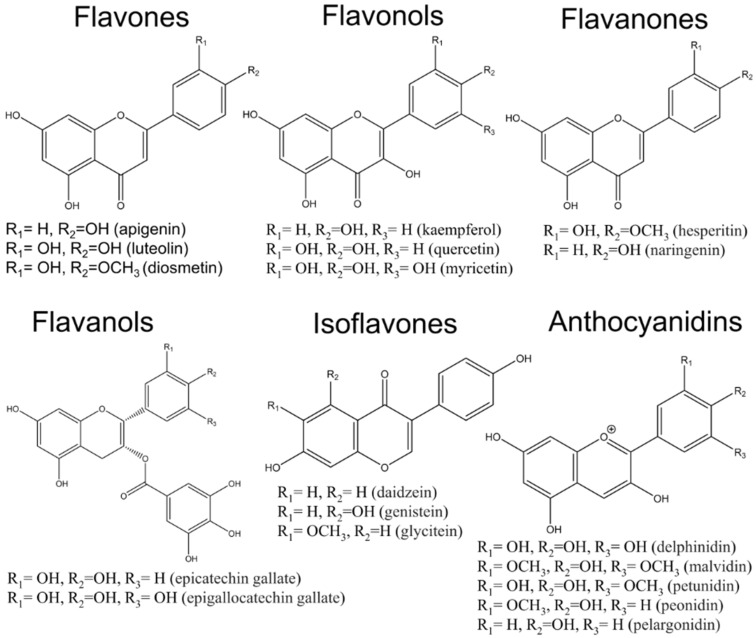
Chemical structure of the main flavonoids.

Phytochemicals are natural, plant-derived compounds that have been used for the treatment of various diseases, including cancer. In vitro and in vivo studies have demonstrated their influence on tumor proliferation, growth, and metastasis [11,12]. Furthermore, the uses of natural compounds are sustained by their wide availability, high tolerance, and cost-effectiveness when compared to synthetic molecules [13,14]. More than 8000 different compounds of polyphenols (phenolic acids, flavonoids, stilbenes, and lignans) are retrieved from natural sources (fruits, vegetables, and seeds) [15]. Flavonoids belong to the polyphenol class of phytochemicals that include over 4000 members. They have been classified according to their molecular structure that consist of two benzene rings joined by a linear three-carbon chain and form an oxygenated heterocycle (C6-C3-C6) and their large number of functional groups (hydroxyl, methoxyl, and O-glycoside) on the basic benzo-pyrone (C6-C3-C6) structure [16]. Recent attention has focused on the beneficial actions of natural flavonoids (subdivided as flavones, flavonols, flavanones, flavanols, anthocyanins and isoflavones) [17,18]. Since flavonoids incorporate a similar functional structure, the functional diversity is generally attributed to the substituent groups [19], the chemical structure being presented in Figure 1. Flavonoids exert innumerable beneficial effects on human health and are considered to be a molecular template for the design of novel therapeutic agents for various diseases, including lung cancer. Flavonoids’ biological effect initially was mainly attributed to their capacity to inhibit reactive oxygen species (ROS) production, a fact that involved a wide range of key cellular processes, affecting several molecular mechanisms altered in tumor cells [12,18]. Regarding the association between flavonoids and lung cancer risk, small beneficial effects were observed, especially among never-smokers patients [17].

Presently, a single clinical trial on 37 patients with stage III lung cancer was undertaken. In this study, epigallocatechin gallate (EGCG) was given in the concentration of 440 μmol/L during radiotherapy and two weeks after radiotherapy. After the treatment, it was observed that the radiation therapy oncology group (RTOG) score decreased significantly and the pain score of each week was significantly lower than the baseline (ClinicalTrials.gov Identifier: NCT02577393). The same results were observed on patients who received oral Polyphenon E twice daily for 3 months in the absence of disease progression or unacceptable toxicity (ClinicalTrials.gov Identifier: NCT00611650).

The purpose of this review is to cluster all the latest investigation presented in the literature concerning the biological effects of natural flavonoids on lung cancer prevention, molecular signaling and therapeutic perspectives, considering the reduced clinical trials for lung cancer.

## 2. Anticancer Effects of Flavonoids in Lung Cancer

**Figure 2:**
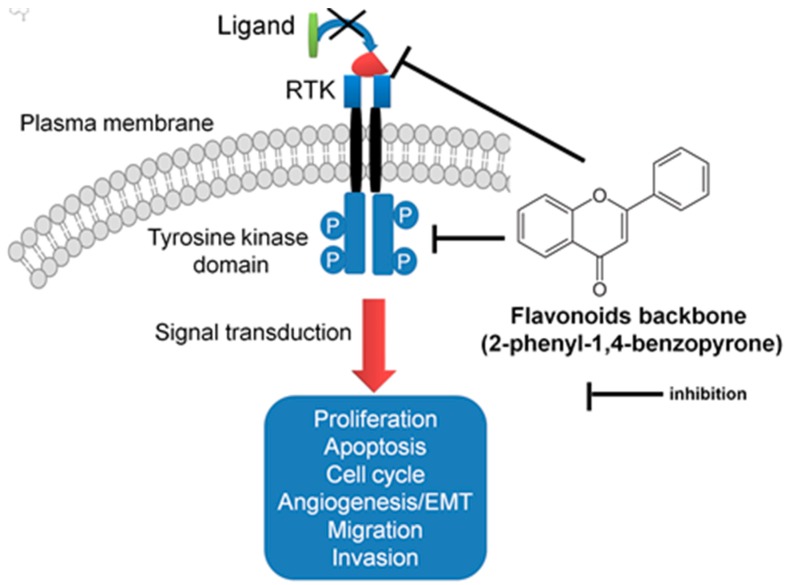
Schematic representation of the mechanism of action of flavonoids as RTK inhibitors, by inhibiting key signal transduction pathways involved in lung cancer.

Dietary flavonoids are natural compounds being used for many years as nutraceuticals due to their numerous favorable properties on human health. This class of compounds is highly prevalent in fruits, vegetables, whole grains, and plant extracts. Flavonoids are the greatest class of polyphenols responsible for the plant pigmentation. Apart from the color, flavonoids have been proved to be responsible for a wide range biochemical functions in seed maturation, protection from different biotic/abiotic stresses, and heat acclimation and freezing tolerance. Flavonoids were developed as a detoxifying and defensive system in plants [20], and in mammalians cells, they have a wide range of biological effects presented in Figure 2 and Table 1.

Flavones are commonly found in many fruits and plant foods [21]. The main flavones are apigenin, luteolin and diosmetin which showed potent inhibitory effects on the proliferation, activation of apoptosis and cell cycle regulation, but also invasion and metastasis [21]. Dietary flavones may serve as preventive/therapeutic agents against different human cancers due to their interfering capacity with epigenetic pathways [22].

Flavonols were proved to have important antiproliferative effects [23], apoptosis also affecting tight junction protein key elements of carcinogenesis [24], invasion and metastatic processes [25].

Flavanones, including hesperidin and naringin, are retrieved in high concentration in citrus, the main biological effects being related to the anti-inflammatory effects [26]. Furthermore, flavanone derivatives have been shown to play a critical role in the cell cycle regulatory proteins expression control [27].

Flavan-3-ols, also found in the literature as catechins, are found in green tea, apples, cocoa, red wine, grapes, and other fruits [16]. Flavan-3-ols have a wide range of antitumoral effects, due to their capacity to regulate NFκB (nuclear factor-κB), MAPK (mitogen activated protein kinases) or PI3K/Akt signaling, target RTK receptors or pro-angiogenic effectors [28]. In vitro and in vivo studies demonstrated that quercetin target aurora B kinase directly affected lung cancer cells by the inhibition of proliferation [23].

Isoflavones (genistein and daidzein) are abundant in soybeans derivative products and are known as RTK inhibitors, targeting the epidermal growth factor (EGF) and interfering with the cell proliferation, migration, and invasion mechanism [29]. Most of the studies on isoflavones are related to the antitumoral effects in hormonal dependent-cancer [19].

Anthocyanidins are common plant pigments responsible for the blue, purple and red colors and have been associated with many biological activities. Anthocyanidins received attention due to their anti-inflammatory, antioxidant, and cancer-inhibitory properties [30]. Anthocyanidins reduce the inflammation by the inhibition of NFκB and Wnt signaling, activate mitochondrial apoptosis. Further, they have been proved to be important regulators of the inhibit Akt/mTOR pathway [30].

## 3. Flavonoids as Key Gene Expression Regulators in Lung Cancer

A number of research studies involving flavonoids provided their role in the prevention and treatment of lung cancer, and have highlighted some of their complex molecular mechanisms of action and potential targets. A couple of important mechanisms comprise of the modulation of carcinogen-metabolizing enzymes, specific cell-cycle arrest and the activation of apoptosis, by the modulation of cell-signaling pathways and the inhibition of the activation of key transcription factors, summarized in Table 2. These mechanisms provide a direct consequence for the inhibition of lung cancer development or progression [38,39]. All these facts suggest the important preventive/therapeutic actions of flavonoids in lung cancer [40].

### 3.1. Flavonoids Interfere with Receptor Tyrosine Kinases Cascade in Lung Cancer

**Figure 3:**
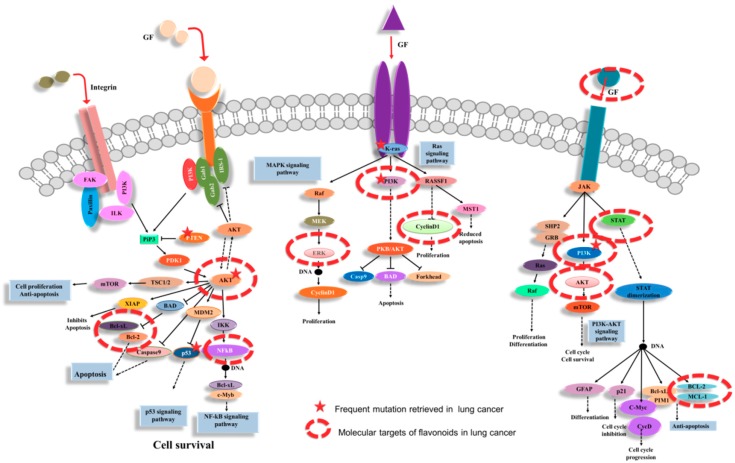
The modulation of cellular signaling pathways by flavonoids, targeting multiple cellular components altered in lung cancer protein kinases C (PKCs) via integrin as an important step in the regulation of cell proliferation and cellular adhesion. Further, RTK interacts with MAPK, NFkB PI3K/Akt and targets of STAT and anti-apoptotic proteins, emphasizing the complex compensatory alterations in lung cancer.

RTKs are a family of cell-surface receptors, highly activated in tumoral pathology. The mutations that affect RTK signaling or downstream effectors conduct the cell transformation, which are frequently retrieved in solid tumors [41]. This makes them important therapeutic targets, including lung cancer. RTK act as receptors for growth factors, hormones, cytokines/chemokine and other extracellular signaling molecules [41]. RTKs activate signal transduction cascade, being able to mediate key signaling pathways, contribute the regulation of cell proliferation, differentiation, survival and cell migration. Flavonoids have been proved to have the capacity to interfere in this signal transduction cascade (Figure 3).

Tumor growth can originate from a high rate of cell division and/or a reduction in the rate of cell death. Flavonoids can affect both of these processes, interfering with key regulatory effectors. Flavonoids are presented in the literature as protein kinase inhibitors for cancer. This was demonstrated by studies proving the direct binding or by molecular modeling [40]. The effect on this kinase function might be independent to the classical antioxidant effect observed [40], but at the same time the reactive oxygen species (ROS) activated MAPK (mitogen-activated protein kinase) cascade, Janus kinase/signal transducers and the activators of transcription (JAK/STAT) pathways or PI3K (phosphatidylinositol 3-kinase)/Akt (serine/threonine kinase 1) [42,43,44].

It is well known that the main barrier in cancer treatment is related to the activation of MAPK in a direct relationship with the persistent activation of transcription factors like NFκB (nuclear factor-κB) or AP1 (activator protein 1) [43]. The activation of NFκB often occurs in lung cancer and contributes to aggressive tumor growth and resistance to chemotherapy and radiotherapy. This factor has proven to be specifically inhibited by dietary flavonoids. After short-term EGCG, the exposure of lung cancer cells was observed in that EGF-induced EGFR, Akt and ERK1/2 activation was substantially decreased [45]. In vitro studies demonstrated that fisetin inhibited the growth and migration of non-small cell lung cancer by inhibiting the activation of the ERK signaling pathway via MEK1/2 [46].

An important molecular target regulated by dietary flavonoids is represented by the PI3K pathway. This signal transduction pathway actively participates in the regulation of cell proliferation and survival, differentiation regulation, cellular adhesion, cell motility, and invasion [31]. The targeting of the PI3K/Akt pathway might be an attractive therapeutic strategy to overcome the struggle of the clinical challenges of lung cancer tumor heterogeneity and acquired resistance, being important members of the flavonoids that are represented by apigenin and lutein [31]. Lutein inhibits the PI3K/Akt signaling pathway, leading to a reduced cell proliferation and activation of apoptosis in lung cancer cells [42].

The Akt proteins are serine/threonine kinases that function as leading regulators of cellular proliferation and apoptosis. The important role of AKT was demonstrated by multiple studies that discussed their development in lung cancer [42,47]. Apigenin was demonstrated to be a new inhibitor of AKT in lung cancer suppressing phosphorylation of Akt and inhibited the gene expression of MMP-9 (matrix metalloproteinases-9), GSK-3 (glycogen synthase kinase-3β), and HEF1 (human enhancer of filamentation 1) [31]. Naringenin treatment showed significant alteration in lung cancer cell proliferation by the inhibition of AKT and MMP2/9 activities in a dose-dependent manner [48].

JAK-STAT3 signaling is activated by the effect of targeting the downstream cytokine receptors, with an impact on a wide range of cellular functions being interrelated with MAPK effectors, Akt or the proteins regulated by cell death machinery (proapototic protein BAD or caspases) which promotes cell survival [40]. Kaempferol and luteinol decrease claudin-2 expression in A549 cells by the inhibition of the interaction between STAT3 and the promoter region of claudin-2, indicating that kaempferol may directly block the interaction of STAT3 on DNA [24]. Daidzein action was mediated by restoring the STK4-induced YAP1 phosphorylation, and the components of Hippo pathway STK4 with significant inhibition of lung cancer cells [49]. A549 and NCI-H358 NSCLC cells after treatment with quercetin have resulted in significant increases of the apoptotic cell population and caspase-3 activity and the loss of MMP in a time- and dose-dependent manner [50].

### 3.2. Flavomoids Affects Cell Proliferation, Apoptosis and Autophagy 

In addition to the repressive effect on cellular proliferation, flavonoids also enhance the rate of cancer cell death [51] by activating apoptosis and autophagy related mechanisms [52]. An important effector is represented by TP53. At the same time, TP53 is one of the most frequently mutated genes in lung cancer [6], being the most extensively studied and related to the inhibition of cell growth and the induction of apoptosis [6,53]. Posttranslational modification has a crucial role in the p53 function [54]. By studying the cisplatin cytotoxic effect on lung cancer cells, researchers have demonstrated that dietary flavonoids, like apigenin, significantly enhanced p53 phosphorylation [55]. Since apigenin treatment promoted MAPK activation, the increased p53 phosphorylation was revealed to be modulated by MAPK, with the essential role in p53 accumulation and the proapoptotic effect. Furthermore, apigenin was observed to amplify the inhibitory effect on cisplatin proliferation in A549 wild-type p53 cells. This effect was not found in the H1299 p53-null cells. Apoptosis was induced by flavonoids in mice lung tissues injected with A549 cells through the caspase-3 and TP53 pathway, p-TP53 and BAX expression which increased due to flavonoid treatment [56]. Additionally, apigenin significantly increased the expression of p53 by suppressing the phosphorylation of IκBα and p65 nuclear translocation [57]. Studies on the effect of quercetin in A549 cells and H1299 cells observed induced-treatment apoptosis in a dose-dependent manner. The cytotoxicity increased after treatment with a specific p53 inhibitor and transfection of a p53 antisense oligodeoxynucleotide [58]. Flavonoids from *G. pentaphyllum* modulated the expression of A, cyclin B and p53 in A549 cells, but not in H460 cells. The difference between p53 expression in the studied cell lines can be explained by the lack of cell cycle arrest in H460 cells [59]. In H522 lung cancer cells, hesperetin increased apoptosis which was correlated with the downregulation of p53 levels [60]. In NSLC A549, luteolin treatment increased the expression of p53 [61,62].

Luteolin inhibited NCI-H460 cell migration in a dose-dependent manner. The anticancer effect of luteolin was induced by Sirt1-mediated activation of the caspase-3 pathway and by the inhibition of the protein expression level of Bad and the Bcl-2/Bax ratio [63]. In lung cancer cells, luteolin acts as a radiosensitizer by increasing apoptotic cell death through the activation of a p38/ROS/caspase cascade [33]. In vitro studies on the effect of naringenin in A549 cells demonstrated significant enhancement of TRAIL-induced apoptosis through the induction of DR5 expression [64]. On H1299 and A549 lung cancer cells, EGCG inhibited the expression of Caspase-3, Bax, and Bcl-2 protein by inhibiting the activation of the PI3K/Akt signaling pathway in a dose-dependent manner [65]. The antiproliferation and pro-apoptotic effects of genistein on A549 lung adenocarcinoma cells is due to the downregulation of Bcl-2 and upregulation of Bax [66].

### 3.3. Flavonoids as Cell Cycle Modulators

The cell cycle is a highly regulated process to ensure proper division of the cell, being supervised by a precise set of proteins that act as checkpoints for correct cell division. The balance between these essential proteins is vital. Flavonoids have been demonstrated to interfere in the regulation of the three main checkpoints (G1, G2 and M). Most literature data reveal that flavonoids target proteins that are implicated in the regulation of the G2/M checkpoint. For example, cyclin-dependent kinases (CDKs) are a group of serine/threonine kinases involved in cancer pathology with essential roles in the apoptosis, differentiation and cell division cycle [67,68]. Genistein significantly inhibited the proliferation and migration of H446 cells inducing apoptosis and G2/M phase cell cycle arrest. Furthermore, genistein treatment demonstrated that FoxM1 may be a novel therapeutic agent down-regulating a series of FoxM1 target genes involved in the cell cycle and apoptosis, including Cdc25B, cyclin B1, and survivin [37]. The flavanones. like hesperetin, have anti-inflammatory and anticancer effects in the A549 cells and inhibit IL-1β-stimulated cell proliferation, COX-2 expression and PGE2 synthesis [69]. Another study on A549 cells reveal that hesperidin have the capacity to induce apoptosis and G0/G1 cell cycle arrest [35].

### 3.4. Flavonoids Regulate Invasion and Metastasis

Flavonoids have proved to be key regulators of epithelial to mesenchymal transition (EMT) and cell migration and invasion. Liu et al. highlighted the modulation of EMT related effectors, as in lung cancer cells, by the EGCG, having the capacity to inhibit the transforming growth factor β (TGFβ) being induced by the EMT mechanism and the inhibition of the phosphorylated form of Smad2 and ERK1/2 [36]. In a study on the apigenin effect on NSCLC cells, an inhibition of the migration/invasion via suppressing the Snail/Slug-mediated EMT has been observed. The invasive ability of NSCLC cells was modulated by the suppressive interplay of Akt and Snail/Slug signaling, harboring different EGFR statuses [32].

In A549 cells, delphinidin has proved to have a potentially new role in anti-angiogenic action. The inhibitory effects of delphinidin on the vascular endothelial growth factor (VEGF) is due to the suppression of the binding of HIF-1 to the HRE promoter with specifically decreasing the CoCl_2_- and EGF-induced HIF-1α protein expression [70]. Furthermore, delphinidin was demonstrated to have an inhibitory effect of EGFR and VEGFR2 in lung cancer cells. The biological active properties may be explained by the simultaneous inhibition of the EGFR and VEGFR2 signaling pathways, and by the activation of PI3K/Akt and MAPKs [71]. In A549 and H1299 cells, baicalein treatment showed it down-regulated Notch1 and hes-1 expression and significantly inhibited the cell invasion and EMT [72]. In A549 lung cancer cells, EGCG treatment determined TGFβ1-mediated EMT inhibition by suppressing the acetylation of Smad2 and Smad3 [73].

## 4. Flavonoids as miRNA Modulators in Lung Cancer

A non-coding RNA (ncRNA) is a functional RNA molecule that is transcribed from DNA but not translated into proteins [74,75]. The noncoding RNAs are involved in many cellular processes, the most important function being to regulate gene expression at the transcriptional and post-transcriptional level [76,77,78]. Many pre-clinical reports have described the opportunity of restoring the expression level for the oncogenic or tumor suppressor transcripts as anticancer therapeutic strategies [74,79]. Based on the earlier studies, the utilization of these therapeutic strategies include the inhibition of oncogenic and restoration of the tumor suppressors transcripts. The effects of flavonoids on the modulation of miRNA expression and its connected target genes level on lung cancer are presented in Table 3.

Dietary flavonoids have been demonstrated to have a role as potential immune modulators because of their lack of adverse effects, low cost and easy administration [62]. The miR-155-inducing signals use the NFκB pathway and regulate the intensity and duration of the immune response [63]. The miR-155, together with miR-9, miR-21, miR-29a, miR-126, miR-146 were included in the so called inflamma-miRs and have been shown to be involved in various pathologies including cancer [64,65] by regulating NFkB signaling. Considering the fact that genistein downregulates the expression of miR155 in breast cancer cells by regulating the miR-155 targets Foxo3, PTEN and p27 expression [80], the targets found also in lung cancer would be interesting to study in the future regarding modulation of miR-155 by flavonoids in lung adenocarcinoma. miR-155 is a transcript that promote lung cancer progress and was proved to be overexpressed [58]. Furthermore, miR-155 is one of the most important miRNAs involved in cancer and biological processes, like inflammation [59]. In NSCLC, miR-155 has been detected to be up-regulated by suggesting an important role as an oncogene and being associated with a poor prognosis [60,61].

The EGCG treatment can adjust miRNAs that play an important role in the MAPK signaling pathway [81]. The miR-98 is a vital miRNA that was observed to inhibit apoptosis, invasion, proliferation and migration in NSCLC cells through the down-regulation of PAK1 expression [82]. The miR-98 was found to be downregulated in A549 cells EGCG-treated and increased the efficacy of cisplatin [83]. The anti-cancer activity of EGCG in lung cancer cells is due to specifically the upregulation of miR-210 which is a major miRNA regulated by HIF-1α [84]. In the EGCG-treated A549 cells, a lower expression of miR-212 was observed. This indicated the fact that A549 cells are resistant to EGCG treatment [81].

MiR-27a was discovered to be an important regulator in many pathological carcinogenesis processes [2] and can be considered a potential target for lung cancer therapy. TheMiR-27a expression levels were activated through a decrease of the MET protein expression levels in lung cancer cells after genistein treatment [85]. The overexpression of miR-27a and the reduction of MET protein expression revealed that genistein has anti-cancer effects on lung cancer cells in a dose-dependent manner [85]. The MiR-27a function as oncogene by regulation of TGFβ signaling pathway by targeting SMAD2/4 [2].

Another prognostic marker in NSCLC is miR-16 [86]. miR-16 was demonstrated to regulate the pro-tumorigenic potential of lung fibroblasts [87]. In lung cancer cells, the decreasing of claudin-2 expression was mediated by the up-regulation of miR-16 expression after quercetin treatment suggesting a flavonoid inhibition effect [67]. Claudin-2 is connected to the upregulation miR-16 but not of miR-15a, miR-15b, miR-195, miR-424, and miR-497 [88]. Quercetin did not inhibit the phosphorylated form of ERK1/2 and Akt. However, it has the capacity to reduce the expression of the tight junction protein, claudin-2. The transcriptional activity of claudin-2 is up-regulated by STAT3 [88]. miR-340 was reported as a novel tumor suppressor in NSCLC [89]. In A549 cells, kaempferol inhibited proliferation and induced apoptosis and autophagy. Kaempferol treatment up-regulated the expression of miR-340. Therefore, an increase of the PTEN level and the decreasing of p-PI3K and p-Akt levels [34] was observed.

## 5. Flavonoids in Combination with Chemotherapeutic and Radiotherapy Treatment in Lung Cancer

Several studies have revealed that these compounds have the capacity to potentiate the anticancer potential for most of the cases [90], supporting normal cells from the secondary effects as a consequence of chemotherapy and radiotherapy [91]. At the same time, they can modulate the inhibition of key multiple singling pathways activated in cancer which evidently provides important benefits in anticancer treatment [76,90]. The standard chemotherapy cytotoxic acts by causing free radicals which can be balanced by natural phytochemicals that have an important role in prevention of this. However, they are mainly used as adjuvant agents. These antioxidant effects can protect from ROS normal cells, but also the tumor cells. This can be two-edged. In some cases, a positive effect has been observed. However, in other cases, this can be related to the diminution in the effectiveness of cytotoxic therapy which is difficult to demonstrate and subtract beyond the side effects observed from chemotherapeutic treatment and radiotherapy [91,92]. The beneficial effects of flavonoids, in combination with chemotherapeutic and radiotherapy treatment in lung cancer, are presented in Table 4.

In the NSCLC cell lines, EGCG acts as an adjuvant to combat cDDP resistance by EGCG-mediated CTR1 mechanism via NEAT1/mir-98-5p crosstalk [93]. In NSCLC cells after treatment with combination of metformin and EGCG, it has been observed that metformin sensitized to the EGCG treatment by suppressing the Nrf2/HO-1 signaling pathway [94].

Quercetin enhances the sensitivity to gemcitabine treatment in lung adenocarcinoma by increasing cells apoptosis via inhibiting HSP70 expression [95]. Simultaneous treatment of resveratrol and clofarabine induced apoptosis in H-2452 cells by reducing Mcl-1 protein level [96]. Co-treatment with resveratrol and erlotinib on lung cancer cells inhibited the Akt/mTOR/S6 kinase pathway enhanced the anti-tumor effects of erlotinib and repressed the expressions of anti-apoptosis proteins [97]. In chemoresistant lung cancer cells, EGCG induces the reversion of cisplatin resistance mediated by downregulation of AXK and TYRO3 receptor tyrosine kinases. After combination treatment with honokiol and cetuximab in non-small cell lung cancer H226 cell line has been observed a downregulation of HER family and their signaling pathways [98].

In lung cancer cells, a combination treatment of quercertin and gemcitabine had significant antiproliferative and pro-apoptotic activities by the downregulation of the HSP70 expression [95]. After the combined treatment of fisetin with paclitaxel in the A549 cells, a relation between the autophagic and apoptotic cell death has not been observed as the percentage of the apoptotic cells did not increase significantly [99]. Since radiotherapy is one of the prime treatment measures for lung cancer, the need to enhance radiotherapy efficacy and protect normal tissues has appeared. The Bcl-xL pathway inhibition has been demonstrated to improve the resistance to radiotherapy in lung cancer patients [100]. Genistein treatment increased the radiosensitivity of lung cancer cells through stimulating apoptosis due to the reducing plasmic Bcl-xL levels [101]. Baicalein increased the sensitivity of cisplatin in lung cancer cells via the PI3K/Akt/NFκB pathway mediated-EMT [102]. The combined treatment of diosmetin and paclitaxel synergistically suppressed lung cancer cells via ROS accumulation through the PI3K/Akt/GSK-3β/Nrf2 pathway disruption [103].

## 6. Conclusions and Perspectives

Flavonoids have received much attention, as demonstrated by the high number of papers published in the last years. Novel mechanistic insights have been demonstrated in cancer therapy where most of the beneficial effects have altered oncogenic signaling, including lung cancer.

The flavonoids discussed in this article inhibited lung carcinogenesis in preclinical studies and have the capacity to target signaling pathways. However, there are many limitations for their use in clinical trials which explains the lack of the data from clinical trials. The main cause is the lack of biomarkers and gaps in our comprehension of the pathogenesis of lung cancer along with lack of good models for risk prediction.

Therefore, a combination of these agents would be more helpful to be used in future clinical trials. For a successful therapy, it would be more reasonable to use the different combined scenarios of therapeutic agents and flavonoids, while also decreasing the dose of chemotherapeutics resulting a decreased in toxicity and providing a maximum efficacy by targeting multiple signaling pathways.

A thorough comprehension of the association between the different functional groups within the structures of flavonoids and their impact on molecular mechanism is vital for the additional advancement and modification of the basic structure of flavonoids in order to increase their therapeutic efficacy. This knowledge will aid in the development of improved therapeutic strategies for the prevention and treatment of solid tumors, including lung cancer. However, there are still many challenges related to the effect of flavonoids, considering the lack of the epidemiological data even though they were proved to have remarkable and beneficial pharmacological effects.

## Figures and Tables

**Table 1:** Anti-lung cancer mechanism modulated by flavonoids.

Class of Flavonois	Representants	Source	Effect on Cells	References
Flavones	Apigenin, luteolin and diosmetin	Parsley, celery leaf, pepper, broccoli	Anti-inflammatory, activation of apoptosis, anti-proliferation, anti-migration, and anti-invasion effects	[21,31,32,33]
Flavonols	kaempherol, quercetin and myricetin	Brussel sprouts, apples, onion, leek and beans	Anti-inflammatory, antiproliferative effect, activation of apoptosis and autophagy cell adhesion, invasion and metastasis	[23,24,25,34]
Flavanons	hesperitin, naringenin	Citrus	Anti-inflammatory effects, Inhibition of cell proliferation, activation of apoptosis	[26,35]
Flavanols	catechin, epicatechin gallate, epigallocatechin, epigallocatechin gallate	apples, pears, grapes, berries green tea and cocoa	suppressing proliferation, inducing apoptosis, inhibition of EMT	[36]
Isoflavons	daidzein, genistein, glycitein	Soy products	Inducing apoptosis, cell cycle arrest, targeting tyrosine kinase inhibitors	[29,37]
Anthocyanidins	delphinidin, malvidin, petunidin, peonidin, pelargonidin	black berries, black currant and blue berries	Anti-inflammatory effect, Inhibition of proliferation, activation of apoptosis	[30]

**Table 2:** Flavonoids tested lung cancer treatment, preclinical studies emphasis on the molecular mechanism of action.

Phycochemical Class	Phytochemicals	Dose	In Vitro Models	End-Point	Effects	Molecular Target	Reference
Flavones	Lutein	20–160 μM	NCI-H460, HEK-293T cell line	Apoptosis assay, western blotting, RT-PCR	Apoptosis activation	Bad↓, Bcl-2↓, Bax↓, caspase-3↓ and Sirt1↓	[63]
		0–100 μM	H1299 and -H460 cells	Immunoblot analysis, PI assay	Apoptosis activation	p38/ROS/caspase cascade↑	[33]
	Apigenin	0–100 μM	A549 cells	MTT, colony assay, Transwell assay, western blot	anti-proliferation, anti-migration, and anti-invasion effects	↓Akt affecting PI3K signaling	[31]
		5–80 μM	A549, H1975, and HCC827 NSCLC cell lines	Transwell migration and invasion assays, RT-PCR	Inhibition of the migration/invasion of NSCLC cells	Akt and Snail/Slug ↑	[32]
	Baicalein	0–100 μM	A549 and H1299 cells	Western blot, QRT-PCR	Inhibition of cell proliferation, down-regulation of Notch1 and hes-1 expression	Cyclin D1 and CDK1↓	[72]
Flavonols	Quercertin	0–200 µM	JB6 Cl41 cells and A549	Anchorage-independent transformation assay, Microscale thermophoresis	Suppression of cells proliferation	Aurora B kinase↓	[23]
		0–200 µM	NCI-H358 and A549 cells.	Apoptosis, microarray	antiproliferative effect	Caspase-3↑	[50]
	Kaempferol	1–50 μM	A549 cell line	MTT test, Transfection, PCR	inhibition of cell proliferation	STAT3↓, claudin-2↓	[24]
	Fisetin	0–40 μM	A549 cells	MTT test, RT-qPCR, flow-cytometry	Apoptosis activation	ERK1/2↓	[46]
Flavnones	Hesperetin	0–100 μM	A549 cells	RT-PCR, western blot	inhibition of cell proliferation	ERK1/2↓, HFKb-p65↓	[69]
		50–125 µg/mL	A549 cells		inhibition of cell proliferation and cell cycle progression		[35]
	Naringenin	0–300 µM	A 549 cells	RT-PCR, western blot	suppression of Akt activity and the downregulation of MMP-2 and -9	Akt↓	[48]
		0–500 µM	A 549 cells	Apoptosis assay, western blot	Cells apoptosis	Bid and DR5↑	[64]
Flavanols	EGCG	0–40 µM	H1299 cells	Cell proliferation, apoptosis assay, western blot	suppressing proliferation, inducing apoptosis	PI3K/Akt↓	[65]
		0–40 µM	A549 cells	Cell culture and transfection, Western blot, Flow cytometry	Decreased EGF-induced EGFR, Akt and ERK1/2 activation.	EGFR↓	[45]
	EGCG	0–40 µM	A549 and NCI-H1299 cells	Scattering assay, wound healing assay, in vitro invasion assay, qRT-PCR, Western blot, confocal microscopy	Cell proliferation, EMT	TGFβ↓, Smad2↓ and Erk1/2↓	[36]
	EGCG	0–100 µM	A549 cells	HAT activity assays, Immunoprecipitation and western blot analysis, RT-PCR	TGF-β1-induced EMT inhibition	TGFβ1↓, Smad2↓, Smad3↓	[73]
Isoflavones	Daidzein	0–60 µM	NSCLC cells	TUNEL assay. Real-time PCR and western blotting	Inducing apoptosis	STK3↓, STK4↓, YAP1↓, caspase3↓	[49]
	Genistein	0–100 µM	H446 cells	Apoptosis assay, colony assay, RT-PCR, western blot	apoptosis and G2/M phase cell cycle arrest	Cdc25B↓, cyclin B1↓, survivin↓	[37]
		0–100 µM	A549 cells	Apoptosis assay, qRT-PCR, Western blot	Inhibition of cell proliferation, cells apoptosis	Bax↑, Bcl-2↓	[66]
Anthocyanidins	Delphinidin	0–80 µM	A549 cells	Cell viability assay, Western blot, RT-PCR, Matrigel plug assay	suppression of the ERK, mTOR, and p70S6K pathways	HIF-1α↓ VEGF↓	[70]
		5–60 µM	NCI-H441, SK-MES-1 and A549	Western blot, Cell viability assay	Apoptosis and angiogenesis	↑caspase-3/9, ↓ anti-apoptotic proteins (Bcl2, Bcl-xL and Mcl-1), ↑pro-apoptotic proteins (Bax and Bak);↓EGFR and VEGFR2	[71]

**Table 3:** Flavonoids as miRNA modulators with implications in lung cancer therapy: Preclinical evidences.

Natural Compound	Preclinical Model	miRNA Targeted	Gene Targeted	Target Mechanism	Technology Approach	References
EGCG (0–50 µM)	CL13 cells, H1299, H460 and A549	miR-210 (↑)	HIF-1α (↓)	reduction of cell growth, hypoxia	Microarray, RT-PCR	[84]
	A549 cells	miR-212 (↓) miR-155 (↑)	MAPK	inhibition of proliferation and migration	NGS	[81]
Quercetin	A549 cells	miR-16 (↑)	Claudin-2 (↓)	Decrease of tight junction mechanisms	qRT-PCR	[88]
Genistein	A549 cells	miR-27a (↓)	MET (↑)	apoptosis and promotes caspase-3/9 activation	Apoptosis assay, western blotting	[85]
Kaempferol	A549	miR-340 (↑)	Cyclin D1 (↓), PTEN (↑)	Cell apoptosis, inhibition of proliferation	Apoptosis assay, qRT-PCR, western blotting	[34]

**Table 4:** The effects of combined lung cancer treatment with flavonoids: Molecular evidences in preclinical studies.

Phytochemicals	Chemotherapeutic	Biological System	Biological Effect	References
EGCG	Cisplatin	A549, H460 and H1299 cells	enhances cisplatin sensitivity, NEAT1 upregulates EGCG-induced CTR1	[93]
	Metformin	A549, H1299 and H460 human NSCLC cell lines	suppressing the Nrf2/HO-1 signaling pathway	[94]
	Cisplatin	H1299 and Lu99 cells	downregulation of AXK and TYRO3 receptor tyrosine kinases	[104]
Quercetin	Gemcitabine	A549 and H460 cells	Inhibition of cancer cell growth and sensitized cancer cells to gemcitabine by quercetin, apoptosis via inhibiting HSP70 expression.	[95]
Fisetin	Paclitaxel	A549 cells	Synergic effect of combination treatment	[99]
Genistein	Radiotherapy	A549 cells	Stimulation of apoptosis, reducing plasmic Bcl-xL levels	[101]
Baicalein	Cisplatin	A549/ /CDDP cells	Induction of apoptosis via PI3K/Akt/NFκB pathway	[102]
Diosmetin	Paclitaxel	A549, H1299, H460, SPC-A1, H441, H1650, Calu-3	Cells apoptosis, increasing paclitaxel efficacy, ROS accumulation, PI3K/Akt/GSK-3β/Nrf2 pathway disruption	[103]
